# Biogeographical Distribution and Community Assembly of Active Protistan Assemblages along an Estuary to a Basin Transect of the Northern South China Sea

**DOI:** 10.3390/microorganisms9020351

**Published:** 2021-02-10

**Authors:** Ran Li, Chen Hu, Jianning Wang, Jun Sun, Ying Wang, Nianzhi Jiao, Dapeng Xu

**Affiliations:** 1 State Key Laboratory of Marine Environmental Science, College of Ocean and Earth Sciences, Xiamen University, Xiamen 361102, China; 22320170154943@stu.xmu.edu.cn (R.L.); 22320150150201@stu.xmu.edu.cn (C.H.); wangjn@xmu.edu.cn (J.W.); wying@xmu.edu.cn (Y.W.); 2 Fujian Key Laboratory of Marine Carbon Sequestration, Xiamen University, Xiamen 361102, China; 3 College of Marine Science and Technology, China University of Geosciences (Wuhan), Wuhan 430000, China; sunjun@cug.edu.cn

**Keywords:** microbial eukaryotes, SSU rRNA, rare, abundant, environmental gradients, driving factors

## Abstract

Marine protists are essential for globally critical biological processes, including the biogeochemical cycles of matter and energy. However, compared with their prokaryotic counterpart, it remains largely unclear how environmental factors determine the diversity and distribution of the active protistan communities on the regional scale. In the present study, the biodiversity, community composition, and potential drivers of the total, abundant, and rare protistan groups were studied using high throughput sequencing on the V9 hyper-variable regions of the small subunit ribosomal RNA (SSU rRNA) along an estuary to basin transect in the northern South China Sea. Overall, Bacillariophyta and Cercozoa were abundant in the surface water; heterotrophic protists including Spirotrichea and marine stramenopiles 3 (MAST-3) were more abundant in the subsurface waters near the heavily urbanized Pearl River estuary; Chlorophyta and Pelagophyceae were abundant at the deep chlorophyll maximum depth, while Hacrobia, Radiolaria, and Excavata were the abundant groups in the deep water. Salinity, followed by water depth, temperature, and other biological factors, were the primary factors controlling the distinct vertical and horizontal distribution of the total and abundant protists. Rare taxa were driven by water depth, followed by temperature, salinity, and the concentrations of PO_4_^3−^. The active protistan communities were mainly driven by dispersal limitation, followed by drift and other ecological processes.

## 1. Introduction

Microorganisms, including bacteria, archaea, protists (microbial eukaryotes), fungi, and viruses, play fundamental ecological roles in marine ecosystems [1]. Protists that play various roles in the aquatic ecosystems (e.g., primary producers, grazers, decomposers, and parasites) are essential for globally critical biological processes, including the biogeochemical cycles, the remineralization of organic matter, and climate regulation [2,3,4]. They have incredibly high abundance and species diversity, which enable them to quickly adapt to the changing surrounding environment [5]. It has been proposed that changes in the taxonomic composition of communities can have substantial impacts on essential ecosystem functions, e.g., primary and secondary production and matter cycling [6]. Consequently, knowledge of the biodiversity, community composition, biogeographical distribution, and the driving factors of protists are critical to understanding the response of marine ecosystems to global changes [1,7].

The abundant (operational taxonomic units, OTUs, with relative abundances > 1%) and rare (OTUs with relative abundances <0.01%) microbial taxa may have distinct characteristics and ecological functions [8,9,10]. The abundant taxa play an essential role in biomass, carbon, and nutrient cycling and are more closely related to other taxa in the ecosystem [11,12,13]. The rare taxa have been proposed to include individuals that grow slowly or remain dormant, contribute predominantly to species richness, and actively maintain ecosystem stability [14,15,16,17,18]. Locally rare taxa can also act as seed bank for seasonal succession or sporadic blooms and respond only when the environment becomes favorable [8,10]. In recent decades, high-throughput sequencing (HTS) on marker genes, e.g., ribosomal genes, has enabled researchers to increasingly discover the enormous diversity of marine microbes, including the rare biosphere with more refined taxonomic resolution [9,19,20,21,22,23,24,25]. However, most studies to date addressing the abundant and rare marine microbial groups focused more on the prokaryotes [26,27,28,29,30,31]. Less attention has been paid to marine protists, leaving the understanding of these two groups largely lagged behind compared with its prokaryotic counterpart [9,14,32,33].

Microbial communities revealed by environmental DNA (eDNA) sequencing may include live, dormant, dead cells, and even extracellular nucleic acids [34,35]. Compared with DNA, extracellular RNA molecules are much less stable and can only survive for much shorter time periods. Thus sequencing based on environmental RNA (eRNA) extracts was proposed to reveal only the metabolically active microbial groups, which has only been recently applied to the study on protists [24,36,37]. To date, most studies focusing on the abundant and rare groups were based on eDNA sequencing [13,25,32]. Only very few studies have investigated these two groups using eRNA-based sequencing, which is even rarer for protists [9,29,38].

The South China Sea (SCS) is one of the largest marginal seas in the western Pacific Ocean [39]. Sharp environmental gradients over small spatial scales have been found along an estuary to basin transect in the northern South China Sea (nSCS) due to the input of freshwater and nutrients from the Pearl River and the intrusion of oceanic water from the SCS: from coastal waters to the open ocean; bottom depths ranging from several tens of meters to over 3000 m; from eutrophic estuary to oligotrophic sea area; low salinity from freshwater to typical oceanic water [40,41]. Furthermore, the viral and bacterial abundances and the chlorophyll concentration showed a nearshore to offshore, surface water to deep water decreasing trend [42,43]. Therefore, the nSCS can serve as an ideal environment for studying the protistan biodiversity, composition, community assembly process, and the underlying control mechanisms.

Previous studies have been done on prokaryotes and viruses, including their biodiversity distribution and community response to environmental factors in the Pearl River Estuary (PRE) and the SCS [44,45,46,47,48,49,50,51]. In terms of protists, several pioneering studies have shown that protists have high diversity in the PRE [52,53,54] and the northern/central SCS [24,55,56], and protistan communities are constrained by complex environmental factors [57,58,59]. However, the composition and assembly processes of the active protistan assemblages along the PRE to the SCS basin transect and the driving factors of the total and abundant/rare taxa need further exploration.

In the present study, using eRNA-based HTS, vertical and horizontal distributions of the diversity, community structure, and assembly processes of the active protistan assemblages along an estuary to basin transect of the nSCS were studied. By measuring multiple environmental parameters, distinct protistan community composition and potential drivers of the total, abundant and rare protistan groups were identified. The active protistan communities were mainly driven by dispersal limitation, followed by drift and other ecological processes.

## 2. Materials and Methods 

### 2.1. Sampling

**Figure 1:**
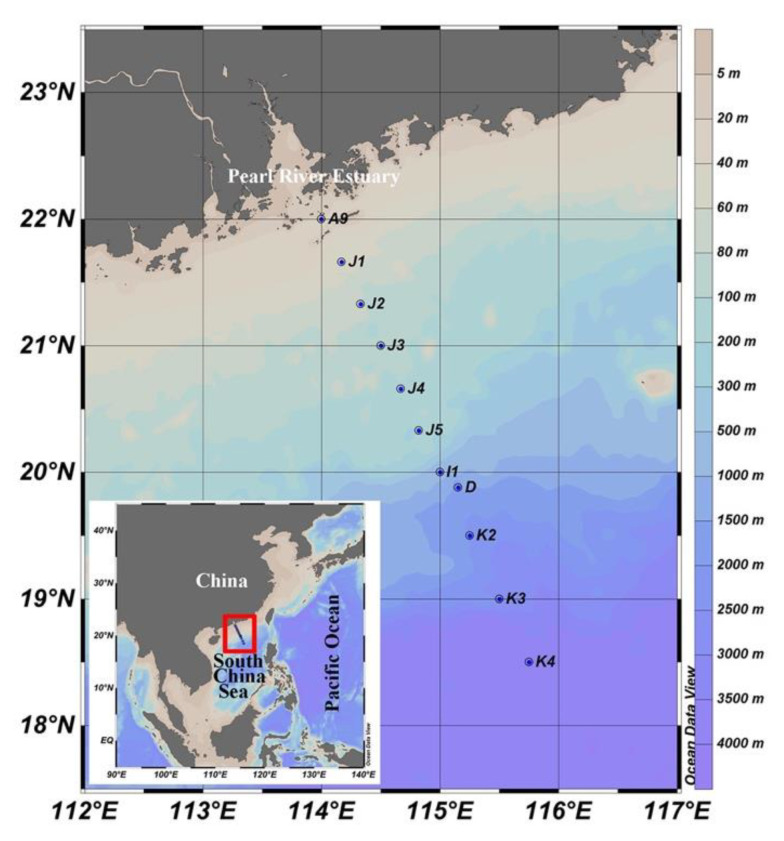
Geographic location of the sampling sites.

Twenty-seven samples from 11 sites with water depth ranging from 5 to 1000 m were collected from 20 August 2014 to 4 September 2014 onboard R/V Dongfanghong II (Figure 1; Table S1). At each sampling site, seawater was collected for the analysis of chlorophyll *a* (Chl *a*), nutrients (including NO_2_^−^, NO_3_^−^, PO_4_^3−^, and SiO_4_^4−^), picoplankton and virus enumeration, and HTS, using Niskin bottles which were set up in a circular rosette attached around CTD sensors (Sea-Bird SBE 911plus, Sea-Bird Electronics, Bellevue, WA, USA). Five replicate samples (2 mL each) of 20 µm mesh prefiltered seawater were fixed with 1% ice-cold glutaraldehyde, flash-frozen in liquid nitrogen, and then stored −80 °C. Two liters of seawater were prefiltered using a 200 μm pore size mesh onto a 0.4 μm pore size filter (Millipore, Burlington, MA, USA), soaked in RNA stabilization solution (Ambion, Austin, TX, USA), and stored at −80 °C for later RNA extraction. Seawater for the determination of Chl *a* were filtered onto a 25 mm diameter Whatman GF/F filter and then kept frozen at −20 °C until analysis. Chl *a* was measured using an acetone extraction method with a Turner-Designs Trilogy^TM^ laboratory fluorometer (Turner Designs, San Jose, CA, USA) [60]. Samples of inorganic nutrient concentrations were frozen, stored at −20 °C and measured by a Technicon AA3 Auto-Analyzer (Bran-Lube, Norderstedt, Germany).

### 2.2. Enumeration of Virus and Picoplankton

The enumeration of viral-like particles (VLPs) and picoplankton was according to [61,62,63]. All samples were added with 1-μm diameter yellow-green fluorescent beads (Molecular Probes, Thermo Fisher, Waltham, MA, USA) as an internal standard with the final concentration of ca. 1% to get a better indication effect. For the enumeration of autotrophic picoplankton including pigmented pico-sized eukaryotes (PPEs), *Synechococcus*, and *Prochlorococcus*, no staining step was performed [63]. Samples for the enumeration of VLPs and heterotrophic prokaryotes were stained with SYBR Green I (Molecular Probes, Thermo Fisher, Waltham, MA, USA) [61,62]. Briefly, for the enumeration of VLPs, after thawed at 37 °C, diluted with 0.02-μm filtered Tris-EDTA buffer (Sigma-Aldrich, Darmstadt, Germany), and stained with SYBR Green I, VLPs were analyzed at a flow rate of 0.1–1 mL h^−1^ and identified on the basis of the green fluorescence and side scatter signal [61]. Autotrophic picoplankton, heterotrophic prokaryotes, and VLPs were analyzed on the same Epics Altra II flow cytometer (Beckman Coulter, Brea, CA, USA). FCS Express V3 software (De Novo Software) was used to obtain VLPs and picoplankton abundance.

### 2.3. High Throughput Sequencing

Environmental RNA was extracted using the RNeasy Mini Kit (Qiagen, Stockach, Germany). Nanodrop ND-2000 Spectrophotometer (Thermo, Waltham, MA, USA) and gel electrophoresis were used to determine the RNA concentration and quality, respectively. Extracted RNA was reverse transcribed using the QuantiTect^®^ Reverse Transcription Kit (Qiagen, China), which removed residual DNA at the first step. The primers (1389F/1510R) were used to amplify the V9 hypervariable regions (ca. 130 bp) of the reverse transcribed SSU rRNA gene [64]. Five individual PCR reactions were performed for each sample and then combined to collect enough amplicons for sequencing. PCR amplicons were purified using Wizard^®^ SV Gel and PCR Clean-Up System (Promega, Beijing, China). Samples were shipped to Majorbio Bio-pharm Biotechnology Co., Ltd. (Shanghai, China) for paired-end (2 × 250 bp) multiplexed sequencing using the Illumina MiSeq platform. All sequence data have been submitted to the NCBI Sequence Read Archive, accessible through the accession number PRJNA687549.

### 2.4. Sequence Processing and Statistical Analyses

Raw reads were screened and assembled using Trimmomatic and Flash software [65,66] and criteria employed as below: (i) reads were truncated at any site that obtained an average quality score of <20 over a 50-bp sliding window and the truncated reads shorter than 50 bp were discarded; (ii) reads with any mismatch in the barcode, more than two nucleotide mismatches in the primer or containing ambiguous characters were removed; and (iii) overlapping sequences shorter than 10 bp or with a mismatch ratio of more than 0.2, were eliminated [58]. Potential chimeric reads were detected and removed in QIIME using USEARCH 6 [67]. Reads were then clustered into Operational Taxonomic Units (OTUs) at ≥97% sequence similarity using UPARSE [68]. The taxonomic assignment was achieved using the BLASTn search against Protist Ribosomal Database 2 (PR2) [69]. OTUs assigned to Bacteria, Archaea, Metazoa, Fungi, and plastids were excluded in downstream analysis. Singletons were also removed before further analysis.

Rarefaction curves were generated using the “rarefaction.single” command in Mothur [70]. Alpha diversity indexes, including OTU richness and Shannon, were calculated based on multiple random resampling at the lowest sequences counts (9634) among samples. A dendrogram was constructed in PRIMER.V.6.0 using the Bray–Curtis similarity index of the normalized data [71,72]. Analysis of similarities (ANOSIM) was used to test sample clustering patterns in PRIMER V.6.0. Similarity percentage (SIMPER) analysis identified OTUs with the most significant differences in community composition among the four groups, which contributed to a total of ca. 50% difference among groups [73,74]. Simple and partial Mantel tests were used to test correlations between environmental variables and communities after 1000 permutations [75]. The paired geographic distances between samples were obtained through the NOAA website (http://www.nhc.noaa.gov/gccalc.shtml, 20 June 2020). 

Quantification of major ecological processes followed [76]. Briefly, two major steps were conducted. We first calculated the β-mean nearest taxon distance (βMNTD) to determine whether communities are under heterogeneous or homogeneous selection [77]. Null models were then constructed using 999 randomizations [76]. Differences between the obtained βMNTD and the mean of the null distribution are denoted as β-Nearest Taxon Index (βNTI). βNTI >2 or < −2 indicates the deterministic processes (i.e., variable selection and homogeneous selection, respectively) while −2 < βNTI < 2 indicates the stochastic processes (i.e., homogenizing dispersal). Second, the Bray–Curtis-based Raup-Crick (RCbray) for pairwise community comparisons were calculated to determine the impacts of dispersal and drift [76]. RCbray > 0.95 indicate dispersal limitation and |βNTI| < 2 and |RCbray| < 0.95 suggest that the community assembly is not dominated by any single process [76].

## 3. Results

### 3.1. Environmental Factors

Temperature and salinity of the surface (5 m) water of the coastal nSCS sites (including A9, J1, J2, and J3; ranging from 28.16 to 29.65 °C, and 31.13 to 33.28, respectively) were significantly lower than the other sites (with mean ± standard deviation of 29.85 ± 0.13 °C and 33.48 ± 0.11, respectively) (Mann–Whitney test, *p* < 0.01). Vertically, water temperature decreased while salinity increased with the increasing water depth (Figure S1; Table S1). The concentrations of nutrients (NO_2_^−^, NO_3_^−^, PO_4_^3−^, and SiO_4_^4−^) showed a distinctly nearshore to offshore decreasing trend horizontally, while a surface to deep water increasing trend vertically (Figure S1, Table S1). The concentrations of Chl *a* of the coastal sites including A9, J1, and J2 were the highest at 5 m, while those of the offshore sites were found at the deep chlorophyll maximum (DCM) layer.

Similar to the physical and chemical parameters of the sampling sites, the spatial distribution of microbial abundances also showed an environmental gradient along the transect (Figure S2, Table S1). The abundances of *Synechococcus* and pigmented pico-sized eukaryotes (PPEs) decreased from 1.00 × 10^5^ to 1.94 × 10^2^ cells mL^−1^ and from 1.51 × 10^4^ to 4.71 × 10^2^ cells mL^−1^, respectively, from the coastal to open ocean sites, while that of *Prochlorococcus* increased from 4.33 × 10^3^ to 1.70 × 10^5^ cells mL^−1^ seaward. The abundances of heterotrophic prokaryotes and VLPs generally decreased both seaward and vertically.

### 3.2. Beta Diversity and Community Composition

**Figure 2:**
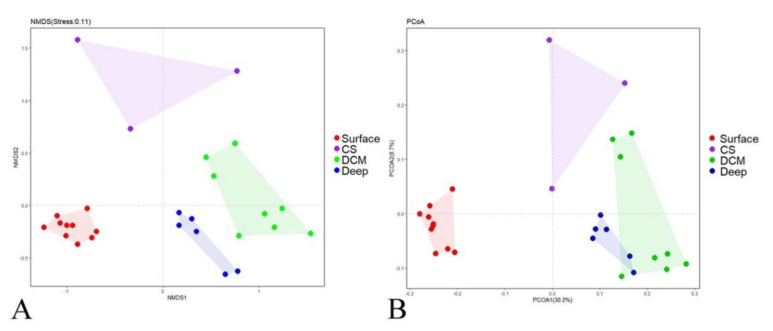
Plots of the nonmetric multidimensional scaling (nMDS) (**A**) ordination and principal coordinate analysis (PCoA); (**B**) based on Bray–Curtis dissimilarities of the protistan communities. Surface, surface waters; CS, coastal subsurface waters; DCM, deep chlorophyll maxima depth; Deep, deep waters.

Although rarefaction analysis showed that protists were not fully sampled in the present study (Figure S3, Good’s coverage ranging from 97.6% to 99.2%), our study gave a snapshot of the active protistan assemblages along an estuary to a basin transect in the nSCS. In the nonmetric multidimensional scaling (nMDS) ordination plot based on the Bray–Curtis dissimilarity of protistan communities, four groups were identified (Figure 2A): (1) Group Surface including all surface samples; (2) Group CS (coastal subsurface water) including samples A9-25m, J1-25m, and J2-25m, which were collected from the subsurface waters near the heavily urbanized PRE; (3) Group DCM including samples collected at the deep chlorophyll maximum (DCM) depth of sites J3, J4, J5, I1, D, K2, K3, and K4; and (4) Group Deep including samples from the deep waters of sites J4, J5, I1, D, K2 and K3. This grouping pattern was statistically supported by the ANOSIM analysis (Table 1). The principal coordinate analysis (PCoA) plot of community taxonomic relatedness quantified by the Bray–Curtis dissimilarity metric also showed a similar clustering pattern (Figure 2B).

**Figure 3:**
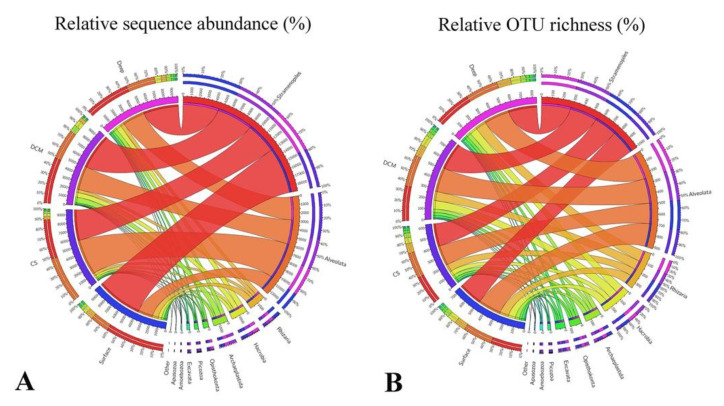
Overview of the relative sequence abundance (**A**) and OTU richness (**B**) of protistan assemblages in Group Surface (surface waters), Group CS (coastal subsurface waters), Group DCM (deep chlorophyll maximum), and Group Deep (deep waters), respectively.

After randomly resampling at the lowest sequence count (9634) among all samples, a total of 3298 OTUs were obtained, ranging from 585 to 977 OTUs per sample. Ninety-seven percent of all retrieved OTUs could be classified at the supergroup taxonomic level (Figure 3). Overall, Group Surface was characterized by having the highest sequences proportions of Stramenopiles (ca. 61%), Rhizaria (ca. 8%), and Picozoa (ca. 2%), and the lowest sequences proportions of Alveolata (ca. 20%). In contrast, Group CS was characterized by having the lowest sequences proportions of Stramenopiles (ca. 41%) and the highest sequences proportions of Alveolata (ca. 48%). Group DCM was characterized by having the highest relative sequence abundance of Archaeplastida (ca. 7%), while group Deep was characterized by having the highest relative sequence abundance of Hacrobia (ca. 7%) and Opisthokonta (ca. 3%). Members affiliated with Excavata, Amoebozoa, and Apusozoa made only minor contributions to the total communities. In terms of OTU richness, OTUs affiliated with Stramenopiles and Alveolata almost contributed equally to the whole community (ca. 34 ± 1% and 31 ± 1%, respectively), followed by Rhizaria (ca. 12%), Hacrobia (ca. 9%), and the other supergroups (Figure 3B).

**Figure 4:**
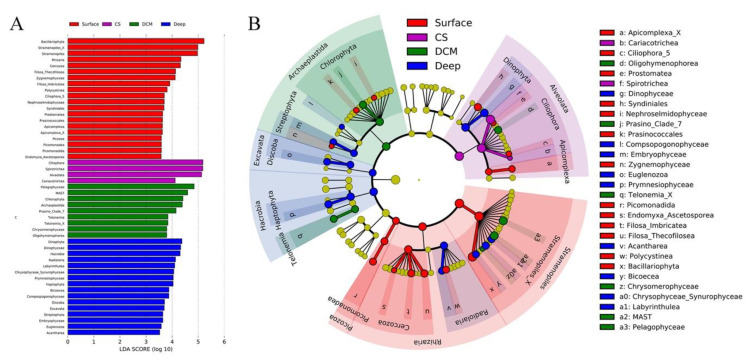
(**A**) Linear discriminant analysis Effect Size (LEfSe) analysis on selected OTUs among the four identified groups, Group Surface (surface waters), Group CS (coastal subsurface waters), Group DCM (deep chlorophyll maximum depth), and Group Deep (deep waters). Only lineages with Least Discriminant Analysis (LDA) values >3.5 are displayed. The multiclass analysis strategy is less strict (one-against-all). Group differences are represented by the color of the most abundant lineages (red: Surface; purple: CS; green: DCM; blue: Deep; and yellow: nonsignificant). (**B**) Cladogram indicating the phylogenetic distribution of the protistan lineages associated with the four groups. Circles indicate phylogenetic levels (from domain to class) in reverse order. The diameter of each circle is proportional to the abundance of the given protistan taxon.

A protistan taxon with a high LDA score in a given group may serve as a potential biomarker for that group [78]. Protistan assemblages at Kingdom, Phylum, and Class taxonomic levels were used to identify the potential biomarkers in the four identified groups, i.e., Surface, CS, DCM, and Deep. Forty-nine protistan assemblages were identified using a logarithmic LDA size effect value of 3.5 (Figure 4A). Cladogram showed that Cercozoa, Bacillariophyta, and Picozoa were more abundant in group Surface. Notably, Bacillariophyta affiliated sequences exceeded 4000 in group Surface (Figure S4). Ciliophora (mainly Spirotrichea) was more abundant in group CS. Chlorophyta (mainly Prosino_Clade_7) and Telonemia were more abundant in group DCM. Radiolaria, Haptophyta (mainly Prymnesiophyceae), Streptophyta, Dinophyta, and Discoba were more abundant in group Deep. At the Kingdom level, seven discriminating lineages among the four groups were revealed by linear discriminant analysis effect size (LEfSe), i.e., Picozoa, Rhizaria, and Stramenopiles for group Surface, Alveolata for group CS, Archaeplastida for group DCM, and Hacrobia and Excavata for group Deep (Figure 4B).

**Figure 5:**
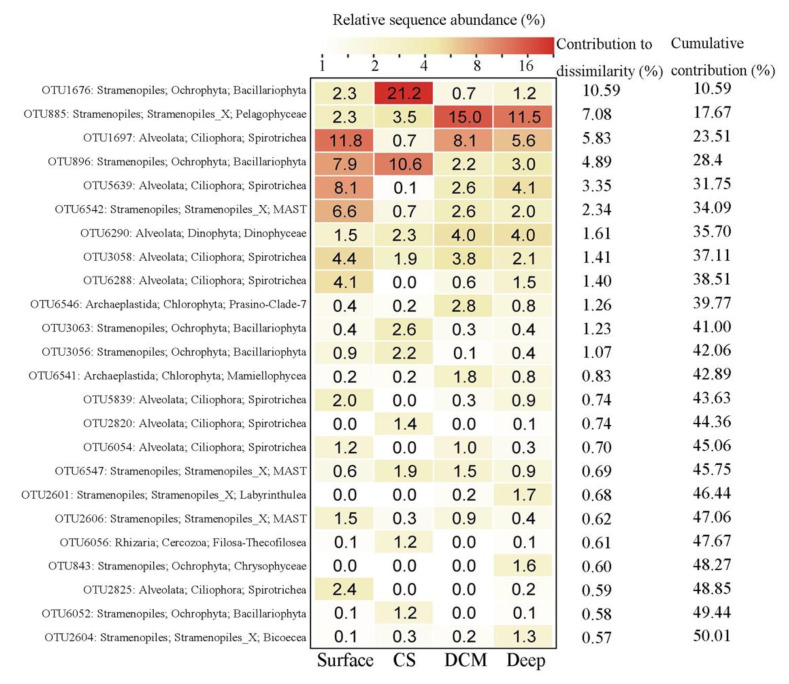
Taxonomic identities of the top 24 OTUs that contributed most to community dissimilarities among the four groups with their relative contributions to each group.

SIMPER analysis identified 24 OTUs that totally contributed ca. 50% of the protistan community dissimilarities among the four groups (Figure 5). These OTUs were affiliated with Stramenopiles (12 OTUs), Alveolata (9 OTUs), Chlorophyta (2 OTUs), and Rhizaria (1 OTU), which contributed ca. 30.9%, 16.4%, 2.1%, and 0.6% of the community dissimilarities, respectively. OTUs identified as members in Stramenopiles were from Bacillariophyta (5 OTUs), MAST (3 OTUs), Bicoecea (1 OTU), Chrysophyceae (1 OTU), Labyrinthulea (1 OTU), and Pelagophyceae (1 OTU). OTUs affiliated with Alveolata were members of the ciliate family Strobilidiidae (8 OTUs) and dinoflagellate class Dinophyceae (1 OTU). The 2 OTUs identified as Chlorophyta were affiliated with Mamiellophyceae and Prasino-Clade-7, respectively. The OTU identified as Rhizaria belonged to Filosa-Thecofilosea (Cercozoa).

### 3.3. Alpha Diversity and Its Driving Factors

The alpha diversity indexes, including OTU richness and Shannon, were comparable in groups Surface and CS that were overall lower than those of the groups DCM and Deep, the latter of which had the highest diversity estimates (Figure S5). Spearman correlation coefficients between the alpha diversity estimates and environmental variables showed that OTU richness was significantly correlated with water depth, followed by the abundance of *Synechococcus*, salinity, latitude, longitude, and the abundance of heterotrophic prokaryotes (Table 2). The Shannon index was significantly correlated with latitude and longitude, followed by the abundance of heterotrophic prokaryotes, water depth, the abundance of VLPs, salinity, and temperature (Table 2).

### 3.4. Abundant and Rare Groups

**Figure 6:**
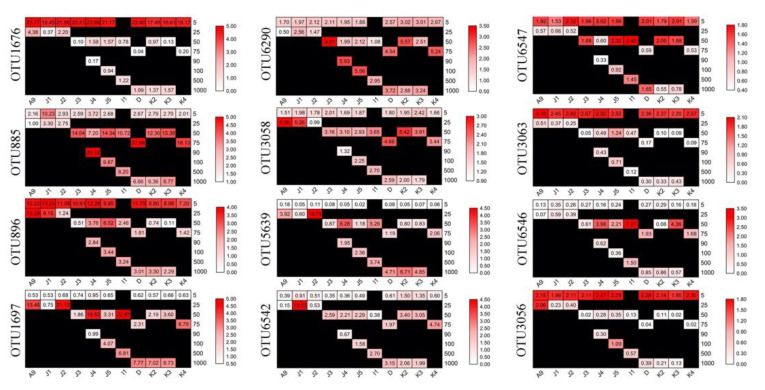
Heatmap showing changes of the relative sequence abundance of the 12 abundant OTUs.

Twelve OTUs were found to be abundant (defined as OTUs accounting for >1% of the total sequences), which totally accounted for ca. 43.9% of sequences and ca. 0.4% of OTUs obtained (Figure S6A). These OTUs were affiliated with Bacillariophyta (OTU1676, OTU896, OTU3063, and OTU3056), Ciliophora (OTU1697, OTU3058, and OTU5639), Pelagophyceae (OTU885), Dinophyceae (OTU6290), Solenicola (OTU6542), Labyrinthulomycetes (OTU6547), and Chlorophyta (OTU6546), which contributed ca. 16.93%, 10.54%, 8.54%, 3.11%, 2.11%, 1.44%, and 1.12% of the total sequences, respectively (Table S2). These abundant OTUs showed distinct distribution patterns. OTU885, OTU896, OTU6290, and OTU1767, were abundant in all samples, with only a few exceptions in some samples (Figure 6). OTUs, including OTU1697, OTU5639, and OTU6542, were intermedium (relative sequence abundance 0.01–1% within a sample) in surface samples and abundant in the subsurface and deep samples, respectively. OTU6546 was found to be intermedium in both surface and deep samples and were only abundant in a few subsurface samples. OTU3063 and OTU3056 were abundant in all surface samples and the intermedium in the subsurface and deep samples (Figure 6).

To show the identity of these OTUs, similarities were obtained between the representative sequences of each OTU with its first BLAST hit (the nearest neighbors, NNs), as well as the first BLAST hit with a species name (the nearest named neighbors, NNNs) in GenBank. High similarities were found between the representative sequences of OTUs and their NN, which were all environmental sequences, 10 of which were identical, and the rest had >99% similarity (Table S2). The high similarities between the representative sequences of OTUs and their NNs indicate that these OTUs were most likely also recovered from other environments.

A total of 2573 OTUs were considered rare (defined as OTUs accounting for <0.01% of the total sequences), which totally accounted for ca. 78.1% of OTUs and ca. 5.5% of sequences recovered. The rare group was dominated by members in Alveolata (ca. 31.6% of the total OTUs), followed by members in Stramenopiles and Rhizaria (ca. 17.3% and ca. 12.3% of the total OTUs, respectively) (Figure S6B). Fifty OTUs were found to be rare in at least one sample (defined as <0.1% of sequences in a sample) but changed to abundant (defined as >1% of sequences in a sample) in at least one sample showing the shift between the locally rare and abundant OTUs. These OTUs were from a diverse of protistan groups including mainly Bacillariophyta in group Surface, Ciliophora in group CS, Archaeplastida in group DCM, and Bacillariophyta and Ciliophora in group Deep (Figure S7).

### 3.5. Effects of Environmental Parameters on the Total (TG), Abundant (AG) and Rare (RG) Groups

In the simple mantel tests, the TG, AG, and RG were significantly correlated with salinity, water depth, and temperature (Table 3). Partial Mantel tests showed that salinity was the dominant driving factor on the TG and AG, followed by a combination of water depth and temperature. On the contrary, water depth and temperature were the dominant driving factor on the RG. TG was significantly correlated with geographic distance, latitude, and longitude after controlling for depth. Also, TG and AG were significantly correlated with the concentrate of Chl *a* and the abundances of heterotrophic prokaryotes. TG was also significantly correlated with the abundances of PPEs, even after controlling for water depth. RG was significantly correlated with the concentrate of PO_4_^3−^ (Table 3). In surface water, geographic distance was the dominant driving factor on the TG and AG, while the temperature was the dominant driving factor on the RG (Table 4).

**Figure 7:**
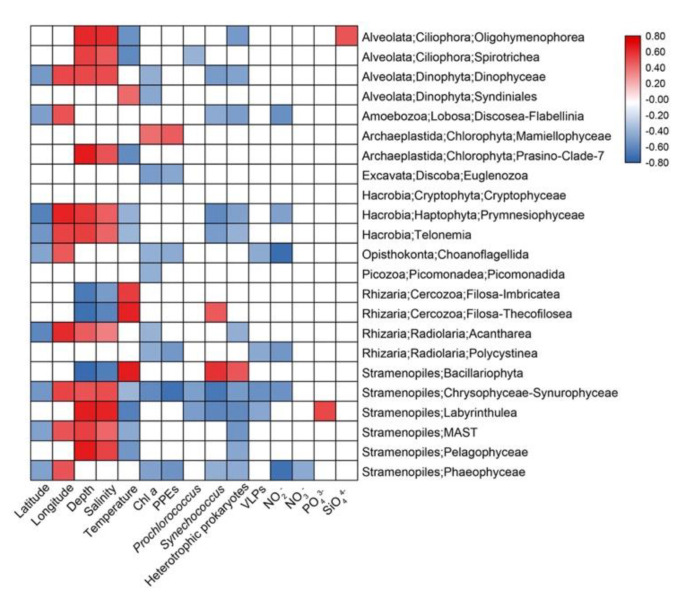
Spearman’s correlations between relative sequence abundance of major protistan groups and environmental parameters. The correlation coefficients’ values are indicated according to the color bar and insignificant values (*p* > 0.05) are left blank.

Spearman’s correlation analyses were conducted to explore the possible influence of environmental variables on the relative sequence abundance of major taxonomic groups (Figure 7). Depth and Salinity were usually positively correlated with several protistan groups (e.g., Ciliophora, Dinophyceae, Haptophyta, MAST, etc.) while temperature usually had the negative effects. The abundance of heterotrophic prokaryotes usually had negative effects on protistan groups including Dinophyceae, Oligohymenophorea, Amoebozoa, Haptophyta, etc. while nutrients only affected a few groups (Figure 7).

### 3.6. The Community Assembly of Protists

**Figure 8:**
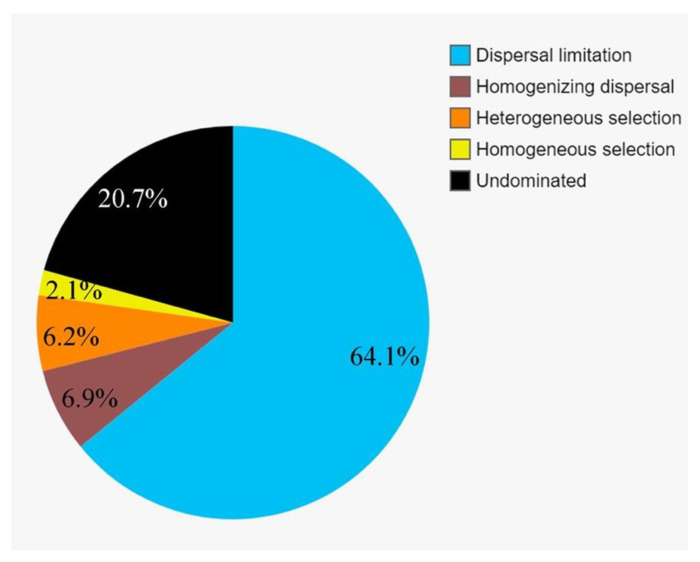
Partition of the community assembly process of the active protistan communities.

To further assess the contributions of spatial and environmental factors on the active protistan communities, quantification of ecological processes mediating community assembly was performed. Dispersal limitation was the primary driver for the community assembly and explained 64.1% of community turnover, followed by drift (ca. 20.7%) and homogeneous selection (ca. 6.9%). The rest processes, including homogenizing selection and heterogeneous selection, totally accounted for ca. 8.3% of community turnover (Figure 8).

## 4. Discussion

### 4.1. Environmental Parameters

The vertical and horizontal distribution patterns of environmental parameters were quite clear. Temperature, the concentration of nutrients and Chl *a*, and the abundances of *Synechococcus*, PPEs, heterotrophic prokaryotes, and VLPs were the highest near the river mouth and decreased as the riverine water mixed with seawater, which was within the range of previous reports [43,47,79,80]. The distribution pattern of the salinity was opposed to temperature. Previous studies showed that nutrients delivered by freshwater input to the estuary were pushed toward high salinity areas [79]. Because the salinity of the eutrophic freshwater of the nearshore PRE was lower than that of the seawater, the surface nutrients of nearshore stations were higher, promoting autotrophic picoplankton growth [79]. Along the vertical way, the temperature gradually decreased, while salinity was opposed within the range of previous reports [24]. The concentrations of NO_2_^-^, NO_3_^-^, PO_4_^3-^ and SiO_4_^4-^ were low in the coastal SCS [47,80] and increased in the open sea along the vertical way, as reported previously [81].

### 4.2. Variations of Major Protistan Assemblages along the Transect

Previous studies investigating protistan diversity were either limited to PRE or specific areas in the SCS, and rather few studies involved large-scale sampling [13,80]. Meanwhile, most of the studies carried out in the SCS focused on protistan diversity in the photic zone, and very few studies involved deep-sea protistan communities [24,55,82]. In the present study, RNA-based HTS that can reduce the interference of dead/dormant cells and extracellular DNA [24] was used to infer how environmental variables drive the vertical and horizontal distribution patterns of the active total, abundant, and rare protistan groups along an estuary to basin transect in the northern SCS. The protistan communities were divided into four distinct groups: Surface, CS, DCM, and Deep (Figure 2). The impact of water depth on the protistan communities was evident, forming groups Surface, CS, DCM, and Deep. Several studies of the protistan community identified water depth as the principal cause of community variability [24,83,84,85]. Group CS clustered sparsely, which showed community composition rapidly changed from the subsurface waters near the heavily urbanized PRE with complex and fast-changing environment conditions [58,79]. 

Bacillariophyta were abundant in group Surface (Figure 4 and Figure S4). The Bacillariophyceae affiliated sequences of the surface samples were high from the PR to the offshore. It has been previously observed that Bacillariophyta is a dominant group, for instance, in the SCS [58] and the northwestern Pacific Ocean [86]. Bacillariophyceae have been considered to be the major source of carbon flux in the surface ocean [87], and many species of Bacillariophyceae showed wide adaptability in the entire freshwater-seawater salinity gradients [88]. Thalassiosirales (Figure 6, OTU1676, and OTU896), which is often associated with eutrophic conditions, occurred mainly at the coastal surface waters [89,90,91]. Rhizosoleniales (Figure 6, OTU3063, and OTU3056) includes many meso- and polyhyaline waters species and may transport a large number of the new nitrogen requirements into the surface waters [92,93]. Cercozoa that included both predatory and parasitic species was reported to have a high diversity of ecological functions in a variety of environments including fresh and sea water, sediment, and soil [94,95,96,97,98]. In the present study, Cercozoa was the dominant group of Rhizaria at the surface water of all stations. Cercozoa sequences were mainly contributed by Filosa-Thecofilosea and Filosa-Imbricatea. Free-living Cercozoa were found to feed on fungi, algae, and other protozoa in a variety of pelagic as well as sediment environments [99]. A previous study has shown that marine cercozoan Crythecomonas was abundant in surface water due to the regulation of stratification process [100].

Spirotrichea (mainly Tintinnida) and MAST-3 were more abundant in group CS (Figure 4 and Figure 6). As members of the microzooplankton, tintinnids are a group of planktonic heterotrophic ciliates that are occasionally capable of preying on most algal production in coastal waters [101]. MAST-3, a group of heterotrophic flagellates and globally widespread bacterial grazers, can significantly affected the subtropical coastal waters ecosystem [102,103,104].

Chlorophyta (mainly Prosino_Clade_7 and Mamiellophyceae) and Pelagophyceae were abundant at the DCM depth (Figure 4, Figure 5, and Figure S4), which is consistent with previous studies in SCS applying restriction fragment length polymorphism (RFLP) and fluorescence in situ hybridization (FISH) approaches [57,80]. A previous study has also shown that Mamiellophyceae dominated the coastal waters of the East China Sea [86]. Pelagophyceae species are found to be key members of the PPEs assemblages at the DCM depth in the NW Pacific [86], the eastern Pacific Ocean [105], the southern Pacific Ocean [106], the northwestern Sargasso Sea [107], and offshore regions of the northern Iberian Peninsula during summer stratification [108]. 

Hacrobia (mainly Prymnesiophyceae), Radiolaria, and Excavata (mainly Discoba) were abundant in group Deep (Figure 4 and Figure S4). Within Hacrobia, Prymnesiophyeae was the dominant group, which was the only dominant PPEs in the warm pool of the NW Pacific [86] and the SCS [53,57,80]. Prymnesiales that have haptonema and are capable of being mixotrophy might contribute to their adaptability to diverse environments such as in the deep sea [109,110]. They can be incorporated into large aggregates or rapidly-sinking fecal pellets of organisms at the higher trophic level and brought to the deep waters [111,112,113,114]. Radiolaria and Excavata were more abundant in deep waters, consistent with previous reports [24,84,115].

### 4.3. Driving Factors of the Total, Abundant, and Rare Protistan Groups

In this study, we investigated how environmental factors, geographic distance, and depth drive the protistan communities, including the total (TG), abundant (AG), and rare (RG) groups, along an estuary to basin transect in the nSCS (Table 3 and Table 4). Simple- and partial Mantel tests showed that salinity was the most dominant driving factor on the TG and AG (Table 3). Our sampling stations spanned the estuary to the open sea, and the salinity ranged from 31.13 to 34.54 (Figure S1). Previous studies on microbial communities in the PRE showed that salinity was the most important driving factor [47,51,58,116]. After controlling for salinity, TG and AG still showed significant correlations with water depth and temperature, which suggested that water depth and temperature were also important driving factors on the TG and AG. After controlling for water depth, no significant correlation between TG/AG and temperature was found. After controlling for temperature, AG but not TG showed significant correlations with water depth. The above result indicated that the effect of temperature was likely resulted from its co-correlation with water depth [100]. Previous studies found water depth was the major driving factor of marine protistan communities, which is probably due to the fact that depth may serve as a good proxy for many physical and chemical variables in the ocean [14,24,83,84,85].

The TG was significantly correlated with geographic distance after controlling for depth (Table 4). Geographic distances may be the result of long-term slow effects compared to water depth and water mass that significantly affect community structure [81]. The importance of geographical distance in the construction of protistan communities does not eliminate the effect of local characteristics on compositional responses [117]. In the surface waters, both TG and AG were significantly correlated with geographic distance. RG were not correlated with either geographic distance or environmental factors (Table 4). It was proposed that the breadth of the taxon niche is the decisive factor influencing the distribution of taxa, and broad niche classification groups are mainly restricted by diffusion, while low niche classification groups are mainly subject to environmental restrictions [118]. Compared to RG, AG and TG have a broader niche [119]. Meanwhile, the TG and AG have many same species, and there may be under great competitive pressure. The high competition exerting on the active microbial community may lead to a certain degree of dispersion restrictions, leading to a significant distance-decay relationship [81].

On the contrary, water depth and temperature were identified to be the dominant driving factors on the RS, and salinity did not affect the distribution of RG after controlling for depth or temperature (Table 3). This reflected that RG and TG/AG might be shaped by different environmental variables [9,14]. Water depth was an integrated factor in constraining temperature, radiation, pressure, and salinity, and RG might play a strong role in those abiotic selection [14]. RG in different depth layers were influenced by contrasting driving factors [13]. Table 4 showed that the correlations between RG and environmental factors were not significant in the surface waters. AG and RG may have different ecological niches and surviving strategies. The few abundant taxa were proposed to be responsible for most of the biomass and carbon cycling, whereas the rare taxa may be important for the cycling of certain elements [11]. Rare microbial taxa were also regarded as a species bank [119], so the active rare taxa might not be influenced by a single environmental factor.

Overall, TG and AG had potential similar drivers, including salinity, followed by water depth, temperature, geographic distance, and biological factors, while RG was dominated by water depth, followed by temperature. It suggested that AG may serve as biomarkers in different geographic areas. AG responded most strongly to abiotic factors than TG, followed by RG, and the varying degrees of TG, AG, and RG responses to the environmental variables contributed to the stabilization of the total community. Rare taxa may become an abundant member of the community when the favorable environmental conditions emerged or when the abundant taxa decrease drastically or even becomes extinct [9,11].

## Figures and Tables

**Table 1:** ANOSIM tests of the groupings of protistan assemblages. Community turnover was based on the Bray–Curtis distance. Surface, surface waters; CS, coastal subsurface waters; DCM, deep chlorophyll maximum depth; Deep, deep waters.

Pairs	ANOSIM	
	r	*p*
Surface vs. CS	0.900	0.003
Surface vs. DCM	0.996	0.001
Surface vs. Deep	0.997	0.001
CS vs. DCM	0.761	0.006
CS vs. Deep	0.679	0.012
DCM vs. Deep	0.534	0.001

**Table 2:** Spearman’s correlation coefficients between alpha diversity estimates and environmental variables. When the correlation is significant both r- and *p*-values are underlined (*p* < 0.05) or in bold (*p* < 0.01).

Environmental Variables	OTU Richness		Shannon	
	r	*p*	r	*p*
Latitude	−0.454	0.017	**−0.565**	**0.002**
Longitude	0.454	0.017	**0.565**	**0.002**
Depth	0.483	0.011	**0.554**	**0.003**
Temperature	−0.328	0.094	−0.405	0.036
Salinity	0.479	0.011	0.482	0.011
NO_3_^-^	0.130	0.586	0.009	0.97
NO_2_^-^	−0.100	0.722	−0.175	0.534
PO_4_^3-^	0.438	0.09	0.037	0.892
SiO_4_^4-^	0.226	0.325	0.103	0.656
Chl *a*	−0.153	0.507	–0.215	0.349
*Prochlorococcus*, abundance	−0.170	0.461	–0.023	0.922
*Synechococcus*, abundance	−0.482	0.027	–0.292	0.200
PPEs, abundance	–0.027	0.907	–0.207	0.368
Heterotrophic prokaryotes, abundance	−0.416	0.039	**−0.561**	**0.004**
Abundance of VLPs	–0.167	0.470	−0.500	0.021

**Table 3:** Simple and partial Mantel tests for the correlations between environmental factors and protistan community composition. When the correlation is significant both r- and *p*-values are underlined (*p* < 0.05) or in bold (*p* < 0.01). (CF: Control for; Geo_distance, geographic distance; HP: Heterotrophic prokaryotes; PPEs, pigmented pico-sized eukaryotes; VLPs, viral like particles)

Environmental Variables	Simple Mantel							Partial Mantel												
	Total		Abundant		Rare		CF	Total		Abundant		Rare		CF	Total		Abundant		Rare	
	r	*p*	r	*p*	r	*p*		r	*p*	r	*p*	r	*p*		r	*p*	r	*p*	r	*p*
Salinity	**0.387**	**0.001**	**0.426**	**0.001**	**0.144**	**0.005**	T	**0.319**	**0.001**	**0.348**	**0.001**	0.059	0.130	D	**0.335**	**0.001**	**0.368**	**0.001**	0.077	0.073
Depth	**0.276**	**0.001**	**0.326**	**0.001**	**0.254**	**0.001**	S	**0.187**	**0.008**	**0.236**	**0.001**	**0.224**	**0.001**	T	0.081	0.077	0.110	0.026	0.108	0.017
Temperature	**0.266**	**0.001**	**0.310**	**0.001**	**0.231**	**0.001**	S	0.134	0.046	**0.171**	**0.010**	**0.191**	**0.002**	D	0.244	0.244	0.029	0.287	−0.006	0.528
Geo_distance	0.090	0.097	0.047	0.200	0.039	0.237	S	0.065	0.153	0.015	0.334	0.028	0.282	D	0.102	0.047	0.059	0.163	0.048	0.176
Latitude	0.092	0.081	0.047	0.210	0.039	0.237	S	0.067	0.137	0.015	0.399	0.028	0.323	D	0.105	0.042	0.061	0.163	0.048	0.168
Longitude	0.095	0.060	0.053	0.181	0.038	0.222	S	0.072	0.117	0.023	0.308	0.028	0.287	D	0.106	0.051	0.064	0.121	0.046	0.184
NO_3_^-^	0.005	0.455	0.003	0.413	−0.002	0.472	S	−0.221	0.993	−0.245	0.995	−0.113	0.929	D	−0.197	0.989	−0.225	0.997	−0.122	0.968
NO_2_^-^	0.159	0.124	0.085	0.248	−0.001	0.465	S	0.044	0.356	−0.046	0.578	−0.059	0.718	D	0.153	0.156	0.063	0.315	−0.019	0.552
PO_4_^3-^	0.184	0.084	0.211	0.065	0.153	0.049	S	0.079	0.250	0.108	0.191	0.132	0.085	D	−0.171	0.943	−0.144	0.906	0.040	0.302
SO_4_^4-^	0.050	0.304	0.043	0.304	0.008	0.426	S	−0.131	0.909	−0.157	0.964	−0.061	0.798	D	−0.217	0.992	−0.258	1.000	−0.130	0.981
Chl *a*	0.185	0.030	0.180	0.034	0.044	0.246	S	0.110	0.097	0.101	0.112	0.025	0.364	D	0.091	0.144	0.081	0.169	−0.012	0.566
*Prochlorococcus*, abundance	−0.052	0.736	−0.059	0.770	0.021	0.368	S	−0.111	0.940	−0.121	0.983	0.002	0.478	D	0.008	0.415	0.001	0.432	0.052	0.242
*Synechococcus*, abundance	0.060	0.227	0.054	0.239	−0.039	0.736	S	−0.070	0.804	−0.080	0.836	−0.090	0.917	D	0.093	0.137	0.089	0.145	−0.041	0.726
PPEs, abundance	0.156	0.047	0.140	0.057	0.003	0.444	S	0.063	0.185	0.043	0.259	−0.035	0.708	D	0.146	0.043	0.126	0.067	−0.026	0.639
HP, abundance	0.160	0.017	0.140	0.030	0.004	0.464	S	0.028	0.297	−0.009	0.525	−0.057	0.830	D	0.097	0.070	0.066	0.136	−0.055	0.838
VLPs, abundance	0.059	0.204	0.070	0.166	−0.007	0.520	S	0.036	0.287	0.047	0.229	−0.020	0.600	D	−0.036	0.676	−0.032	0.648	−0.080	0.887

**Table 4:** Simple and partial Mantel tests for the correlations between environmental variables and community composition in the surface water. When the correlation is significant both r- and *p*-values are underlined (*p* < 0.05) or in bold (*p* < 0.01). (Geo_distance, geographic distance; HP: Heterotrophic prokaryotes; PPEs, pigmented pico-sized eukaryotes; VLPs, viral like particles).

Environmental Variables	Simple Mantel						Partial Mantel						
	Total		Abundant		Rare		Control for	Total		Abundant		Rare	
	r	*p*	r	*p*	r	*p*		r	*p*	r	*p*	r	*p*
Geo_distance	**0.461**	**0.005**	**0.503**	**0.004**	0.209	0.079							
Latitude	**0.458**	**0.008**	**0.499**	**0.002**	0.201	0.093	Geo_distance	−0.017	0.552	−0.035	0.610	−0.092	0.72
Longitude	0.423	0.012	**0.461**	**0.008**	0.182	0.108	Geo_distance	−0.210	0.909	−0.241	0.959	−0.146	0.821
Salinity	−0.024	0.518	0.093	0.360	0.163	0.075	Geo_distance	−0.224	0.814	−0.103	0.634	0.098	0.158
Temperature	0.282	0.151	0.404	0.060	0.184	0.052	Geo_distance	0.154	0.291	0.291	0.139	0.124	0.116
NO_3_^-^	0.253	0.182	0.371	0.081	0.057	0.305	Geo_distance	0.103	0.319	0.234	0.175	0.000	0.505
NO_2_^-^	0.212	0.301	0.406	0.137	0.127	0.236	Geo_distance	0.166	0.323	0.360	0.187	0.104	0.300
PO_4_^3-^	0.467	0.066	0.593	0.027	−0.033	0.651	Geo_distance	0.429	0.105	0.575	0.032	−0.096	0.836
SiO_4_^4-^	0.483	0.036	0.496	0.017	−0.021	0.592	Geo_distance	0.441	0.052	0.458	0.035	−0.069	0.743
Chl *a*	0.195	0.230	0.321	0.115	0.137	0.183	Geo_distance	−0.002	0.473	0.155	0.255	0.060	0.349
*Prochlorococcus*, abundance	0.122	0.203	0.069	0.323	0.140	0.190	Geo_distance	0.095	0.234	0.031	0.388	0.126	0.191
*Synechococcus*, abundance	−0.052	0.548	0.097	0.314	0.040	0.338	Geo_distance	−0.141	0.688	0.022	0.429	0.008	0.458
PPEs, abundance	0.31	0.129	0.449	0.046	−0.074	0.782	Geo_distance	0.209	0.233	0.368	0.089	−0.143	0.931
HP, abundance	0.107	0.294	0.192	0.220	0.016	0.439	Geo_distance	0.078	0.329	0.176	0.225	−0.001	0.506
VLPs, abundance	−0.167	0.778	−0.097	0.621	−0.017	0.515	Geo_distance	−0.166	0.735	−0.088	0.604	−0.004	0.517

## Data Availability

All sequence data generated in this study have been submitted to the NCBI Sequence Read Archive, accessible through the accession number PRJNA687549.

## Supplementary Materials

The following are available online at https://www.mdpi.com/2076-2607/9/2/351/s1, Figure S1: The physical and chemical parameters along the transect from Pearl River estuary to northern South China Sea, Figure S2: The biological parameters along the transect from Pearl River estuary to northern South China Sea, Figure S3: Rarefaction curves of the samples collected, Figure S4: Relative abundances of sequences and OTUs of the eight supergroups. (A) Taxonomic Stramenopiles; (B) Aveolata; (C) Archaeplastida; (E) Rhizaria; (F) Excavata, Opisthokonta and Picozoa, Figure S5: Alpha diversity estimates of OTU richness (A) and Shannon (B) the four groups (surface, CS, DCM, and Deep). ** *p* < 0.01, * *p* < 0.05, Figure S6: Sequences abundance of the abundant (A) and OTU richness of the rare (B) groups at the supergroup taxonomic level, Figure S7: Compositions of the rare taxa that can shift to abundant taxa in the four groups, Table S1: Name, coordinates, and environmental parameters of the samples. HP: Heterotrophic prokaryotes; PPEs, pigmented pico-sized eukaryotes; VLPs, viral like particles; ND: Not detected; BDL: Below detection limit, Table S2: List of the twelve abundant OTUs with the relative abundance of sequences, taxonomic identification, GenBank accession number and the identification of the nearest named neighbor (NNN), similarity (%-S) with the NNN, and GenBank accession number of the nearest neighbor (NN) and similarity (%-S) with NN.
