# Supplementary material for: Biogeographical Distribution and Community Assembly of Active Protistan Assemblages along an Estuary to a Basin Transect of the Northern South China Sea
Source: Microorganisms. 2021 Feb 10;9(2):351. doi: 10.3390/microorganisms9020351 (PMC7916720; doi:10.3390/microorganisms9020351)
Supplement: Supplementary file 1 [file microorganisms-09-00351-s001.pdf]

Supplementary Material

Supplementary Figures

Figure S1. The physical and chemical parameters along the transect from Pearl River estuary to northern South China Sea.

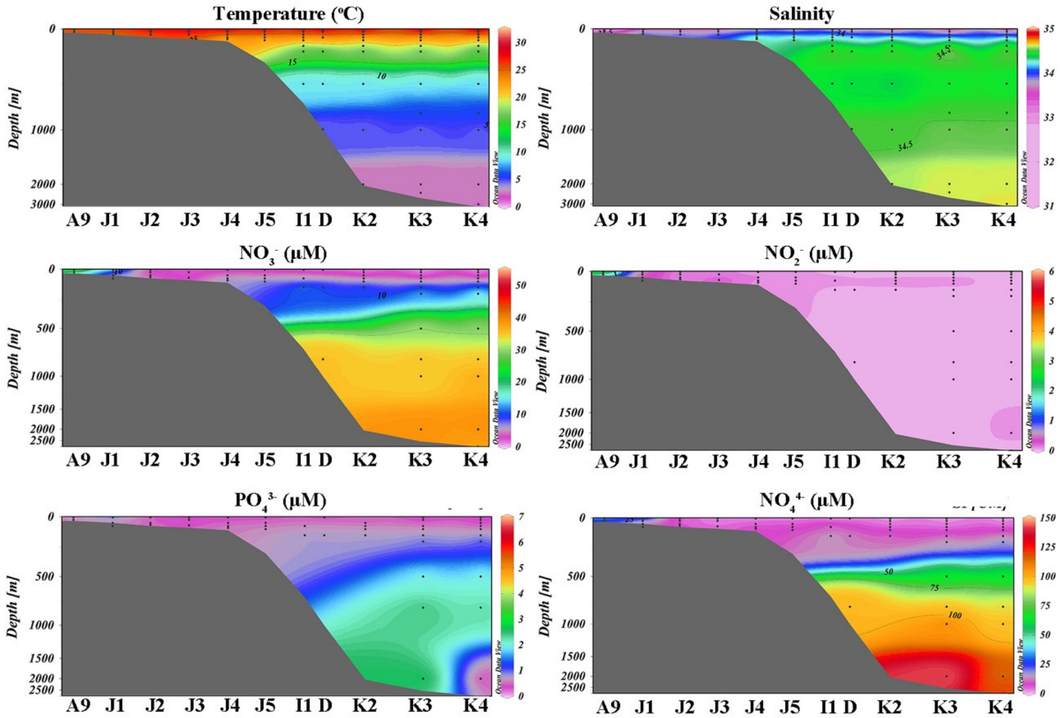

Figure S2. The biological parameters along the transect from Pearl River estuary to northern South China Sea.

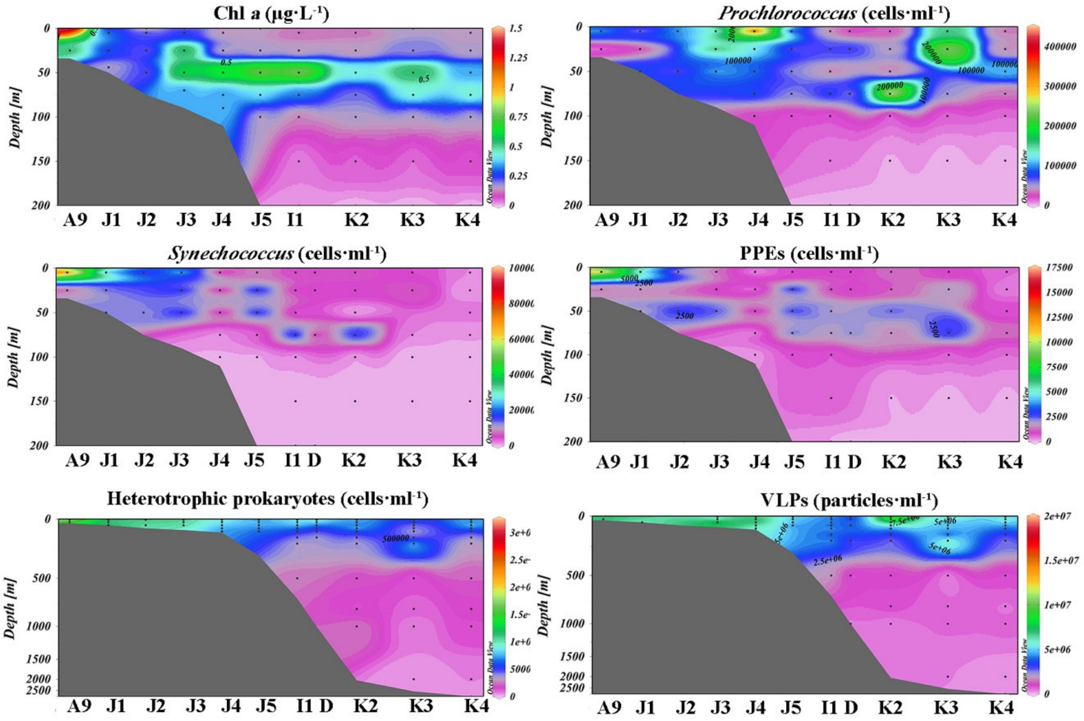

Figure S3. Rarefaction curves of the samples collected.

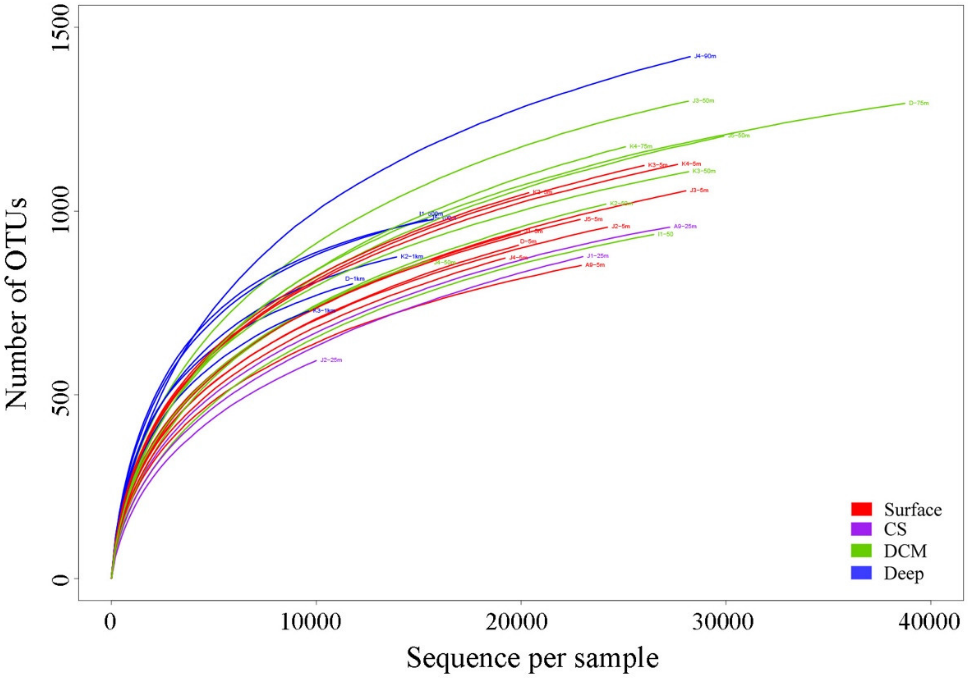

Figure S4. Relative abundances of sequences and OTUs of the eight supergroups. (A) Taxonomic Stramenopiles; (B) Aveolata; (C) Archaeplastida; (E) Rhizaria; (F) Excavata, Opisthokonta and Picozoa.

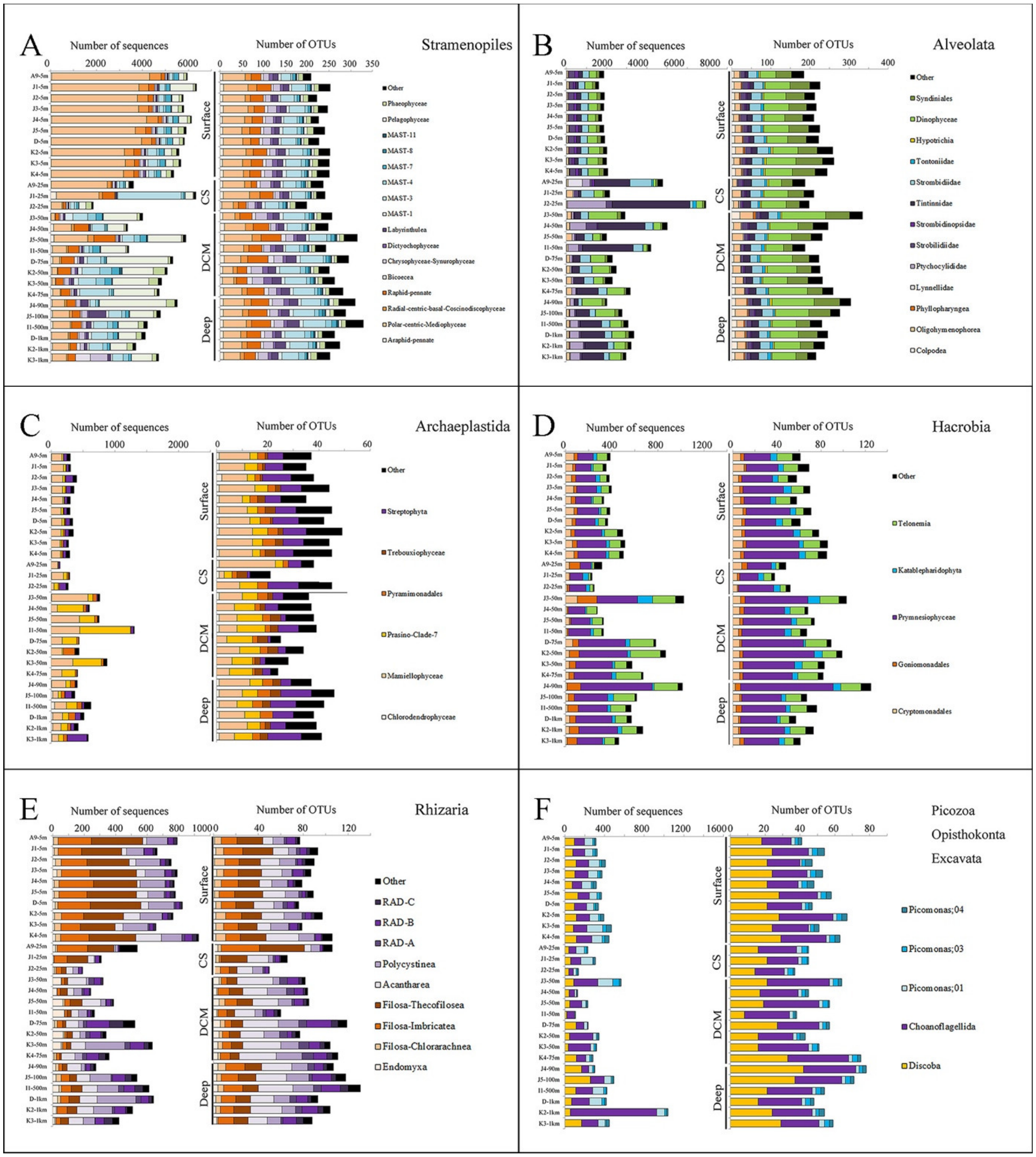

Figure S5. Alpha diversity estimates of OTU richness (A) and Shannon (B) the four groups (surface, CS, DCM, and Deep). ** $p < 0.01$, * $p < 0.05$.

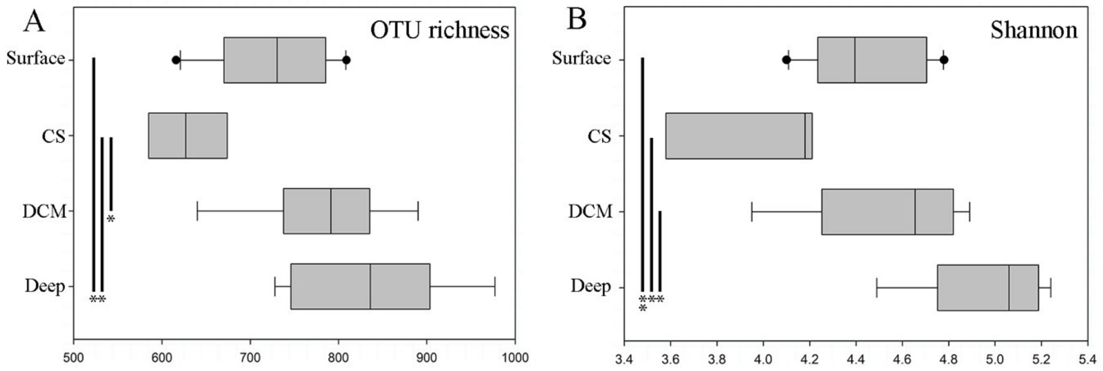

Figure S6. Sequences abundance of the abundant (A) and OTU richness of the rare (B) groups at the supergroup taxonomic level.

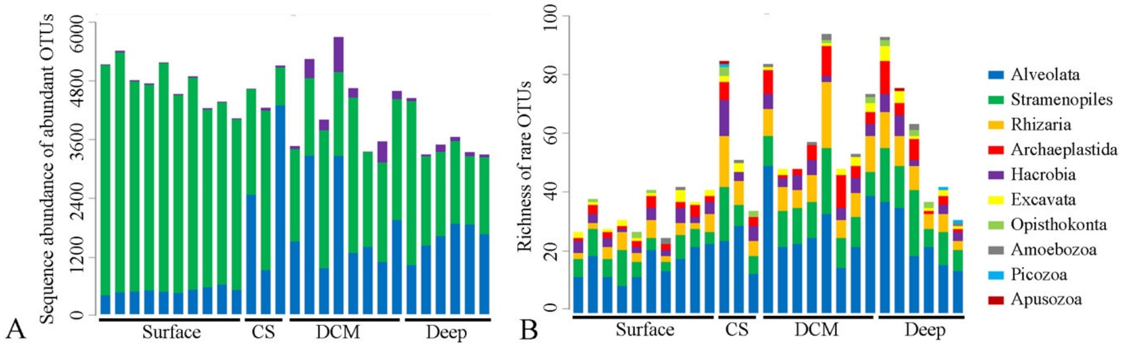

Figure S7. Compositions of the rare taxa that can shift to abundant taxa in the four groups.

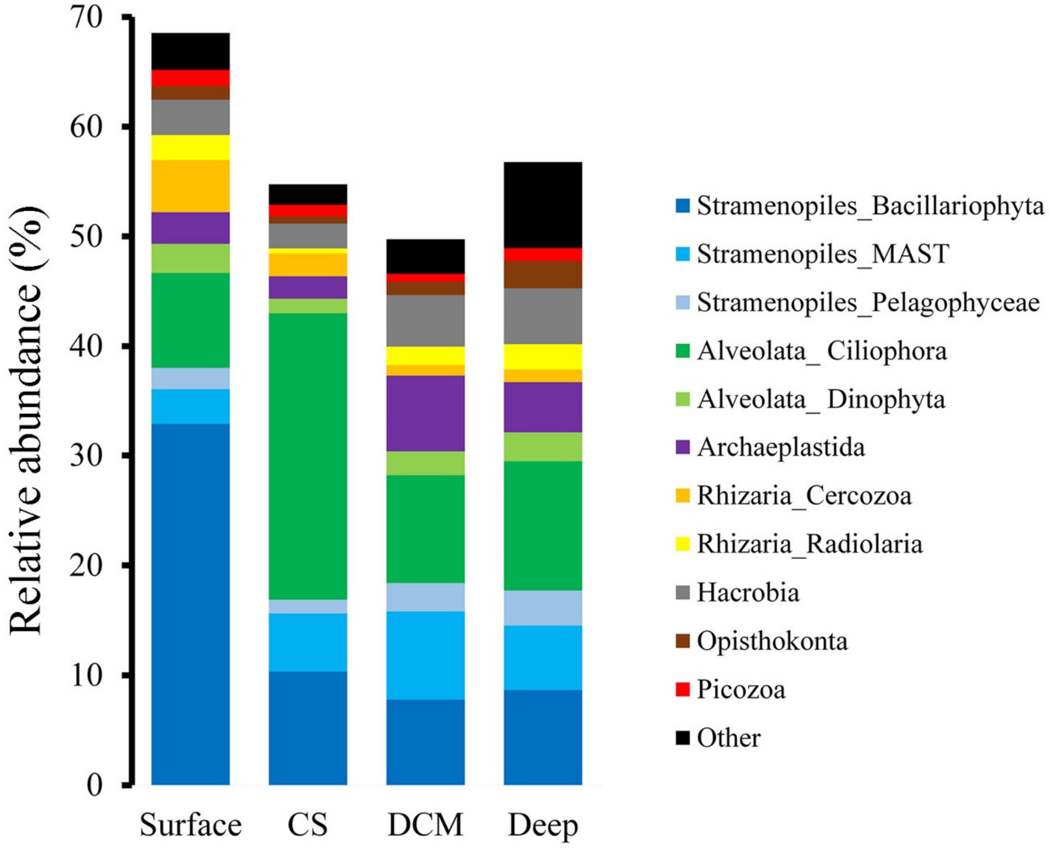

Supplementary Tables

Table S1. Name, coordinates, and environmental parameters of the samples. HP: Heterotrophic prokaryotes; PPEs, pigmented pico-sized eukaryotes; VLPs, viral like particles; ND: Not detected; BDL: Below detection limit.

Sample	Latitude	Longitude	Depth	Depth of sea bottom	Temperature	Salinity	Concentrate					Abundance				
	(N)	(E)	(m)	(m)	(°C)	(PSU)	Chl *a* (μg·L^{-1})	NO_3^- (μM)	NO_2^- (μM)	PO_4^{3-} (μM)	SiO_4^{4-} (μM)	*Prochlorococcus* (cells·ml^{-1})	*Synechococcus* (cells·ml^{-1})	PPEs (cells·ml^{-1})	HP (cells·ml^{-1})	VLPs (particles·ml^{-1})
A9-5m	22	114	5	28	28.16	31.13	1.488	53.608	5.677	0.4	56.242	1.25E+05	1.00E+05	1.51E+04	3.16E+06	ND
A9-25m	22	114	25	28	23.2	34.35	0.526	3.848	0.928	0.428	9.467	4.33E+03	2.20E+03	1.15E+03	1.52E+06	7.59E+06
J1-5m	21.66	114.17	5	46	28.66	33.2	0.296	30.29	0.012	2.138	69.389	1.44E+04	4.80E+03	2.75E+03	6.71E+05	ND
J1-25m	21.66	114.17	25	46	24.28	34.18	0.255	2.171	0.252	ND	4.593	2.24E+04	1.59E+04	1.58E+03	5.97E+05	ND
J2-5m	21.33	114.33	5	71	29.65	33.04	0.237	0.07	0.015	0.183	0.63	1.11E+05	7.60E+03	8.46E+02	1.11E+06	ND
J2-25m	21.33	114.33	25	71	27.53	33.86	0.21	0.068	0.014	ND	0.85	ND	ND	ND	ND	ND
J3-5m	21	114.5	5	76	29.64	33.08	0.267	ND	ND	0.191	0.795	1.05E+05	2.88E+04	1.16E+03	1.26E+06	6.19E+06
J3-50m	21	114.5	50	76	24.87	34.2	0.615	ND	ND	ND	ND	1.16E+05	2.04E+04	2.70E+03	1.22E+06	1.29E+07
J4-5m	20.66	114.67	5	106	30.02	33.69	0.153	0.08	0.013	0.173	0.254	7.14E+04	1.76E+04	9.83E+02	1.04E+06	5.72E+06
J4-50m	20.66	114.67	50	106	24.86	34.21	0.817	3.212	0.159	0.415	3.962	1.05E+05	8.12E+03	2.55E+03	8.40E+05	9.93E+06
J4-90m	20.66	114.67	90	106	19.2	34.51	0.243	9.889	0.043	0.815	11.388	1.69E+04	2.95E+02	1.07E+03	6.53E+05	4.77E+06
J5-5m	20.33	114.82	5	141	29.69	33.49	0.143	0.096	0.015	0.179	0.248	5.52E+04	8.87E+03	8.46E+02	4.52E+05	6.33E+06
J5-50m	20.33	114.82	50	141	24.91	34.15	0.753	1.387	0.162	0.349	2.334	5.06E+04	1.89E+04	2.94E+03	8.96E+05	5.67E+06
J5-100m	20.33	114.82	100	141	18.56	34.54	0.139	10.461	0.035	0.822	10.936	1.15E+04	1.94E+02	6.76E+02	4.76E+05	ND
I1-50m	20	115	50	737	24.21	33.54	0.768	ND	ND	ND	ND	3.51E+04	2.34E+03	1.80E+03	1.21E+06	5.67E+06
I1-500m	20	115	500	737	8.51	34.43	ND	ND	ND	ND	ND	ND	ND	ND	2.33E+05	2.20E+06
D-5m	19.88	115.15	5	1231	29.9	33.5	ND	0.074	0.013	0.16	0.956	1.98E+04	6.50E+03	8.19E+02	1.12E+06	2.97E+06
D-75m	19.88	115.15	75	1231	21.35	34.37	ND	ND	ND	ND	ND	4.65E+04	9.85E+02	2.17E+03	2.88E+05	2.98E+06
D-1km	19.88	115.15	1000	1231	4.51	34.49	ND	ND	ND	ND	ND	ND	ND	ND	ND	4.99E+05
K2-5m	19.5	115.25	5	2176	29.77	33.44	0.12	0.074	0.005	ND	1.065	2.78E+04	5.68E+03	1.27E+03	8.36E+05	7.17E+06
K2-50m	19.5	115.25	50	2176	24.89	34.04	0.419	0.082	BDL	0.188	1.165	3.44E+04	7.81E+02	2.26E+03	5.09E+05	5.37E+06
K2-1km	19.5	115.25	1000	2176	4.59	34.49	ND	ND	ND	ND	ND	ND	ND	ND	2.63E+05	2.55E+05
K3-5m	19	115.5	5	2671	29.78	33.37	0.161	0.074	BDL	0.19	1.288	1.53E+05	4.47E+03	8.57E+02	9.58E+05	5.17E+06
K3-50m	19	115.5	50	2671	24.32	33.98	0.537	0.07	BDL	ND	1.588	ND	ND	ND	2.54E+05	2.72E+05
K3-1km	19	115.5	1000	2671	4.36	34.53	ND	34.472	BDL	ND	108.358	ND	ND	ND	4.37E+04	5.52E+05
K4-5m	18.5	115.75	5	3754	29.95	33.4	0.142	0.168	BDL	0.247	0.947	3.59E+04	1.78E+03	4.71E+02	8.04E+05	4.01E+06
K4-75m	18.5	115.75	75	3754	21.95	34.32	0.456	1.862	0.088	0.389	3.277	3.44E+04	1.17E+03	1.30E+03	7.98E+05	3.06E+06

Table S2 List of the twelve abundant OTUs with the relative abundance of sequences, taxonomic identification, GenBank accession number and the identification of the nearest named neighbor (NNN), similarity (%-S) with the NNN, and GenBank accession number of the nearest neighbor (NN) and similarity (%-S) with NN.

OTU ID	Relative abundance	Group	Name of NNNs and GenBank accession no.	%-S (NNN)	GenBank accession no.	%-S (NN)
OTU1676	8.58%	Stramenopiles; Bacillariophyta; Thalassiosirales	*Skeletonema costatum* (AB948141)	100.00	DQ396523	100.00
OTU885	8.54%	Stramenopiles; Pelagophyceae; Pelagomonadales	*Pelagomonas calceolata* (EF455763)	100.00	KP404872	100.00
OTU896	6.12%	Stramenopiles; Bacillariophyta; Thalassiosirales	*Thalassiosira eccentrica* (JQ217343)	99.23	KX253953	99.23
OTU1697	5.18%	Alveolata; Ciliophora; Tintinnida	*Tintinnopsis radix* (KU715774)	100.00	KX158726	100.00
OTU6290	3.11%	Alveolata; Dinophyceae; Prorocentrales/ Peridiniales/ Gonyaulacales/ Gymnodiniales	*Parvodinium cf. umbonatum* (MG255428)	100.00	KP404853	100.00
OTU3058	2.77%	Alveolata; Ciliophora; Strombidiidae/ Cyrtostrombidiidae	*Apostrombidium orientale* (MH688480)	98.39	KF129744	100.00
OTU5639	2.59%	Alveolata; Ciliophora; Tintinnida	*Favella panamensis* (KU715760)	100.00	KU715760	100.00
OTU6542	2.21%	Stramenopiles; Solenicola	*Solenicola setigera* (HM163291)	92.97	JQ782081	100.00
OTU6547	1.44%	Stramenopiles; Labyrinthulomycetes	*Oblongichytrium sp.* (KY980037)	96.03	JQ781995	100.00
OTU3063	1.19%	Stramenopiles; Bacillariophyta; Rhizosoleniales	*Rhizosolenia setigera* (KY980291)	100.00	GU823605	100.00
OTU6546	1.12%	Viridiplantae; Chlorophyta; Trebouxiophyceae	*Coccomyxa viridis* (HG973007)	90.15	JX188279	100.00
OTU3056	1.04%	Stramenopiles; Bacillariophyta; Rhizosoleniales	*Eucampia sp.* (KX253956)	90.15	KX253956	99.23
